# Virtual Simulation-Based Optimization for Assembly Flow Shop Scheduling Using Migratory Bird Algorithm

**DOI:** 10.3390/biomimetics9090571

**Published:** 2024-09-21

**Authors:** Wen-Bin Zhao, Jun-Han Hu, Zi-Qiao Tang

**Affiliations:** 1 College of Mechanical Engineering, Zhejiang University of Technology, Hangzhou 310023, China; 202005031108@zjut.edu.cn; 2 School of Electronic Information and Electrical Engineering, Chengdu University, Chengdu 610100, China; tangziqiao@cdu.edu.cn

**Keywords:** virtual simulation, hybrid assembly flow shop, multi-mold constraint, migratory bird optimization algorithm, bionic optimization algorithm

## Abstract

As industrial informatization progresses, virtual simulation technologies are increasingly demonstrating their potential in industrial applications. These systems utilize various sensors to capture real-time factory data, which are then transmitted to servers via communication interfaces to construct corresponding digital models. This integration facilitates tasks such as monitoring and prediction, enabling more accurate and convenient production scheduling and forecasting. This is particularly significant for flexible or mixed-flow production modes. Bionic optimization algorithms have demonstrated strong performance in factory scheduling and operations. Centered around these algorithms, researchers have explored various strategies to enhance efficiency and optimize processes within manufacturing environments.This study introduces an efficient migratory bird optimization algorithm designed to address production scheduling challenges in an assembly shop with mold quantity constraints. The research aims to minimize the maximum completion time in a batch flow mixed assembly flow shop scheduling problem, incorporating variable batch partitioning strategies. A tailored virtual simulation framework supports this objective. The algorithm employs a two-stage encoding mechanism for batch partitioning and sequencing, adapted to the unique constraints of each production stage. To enhance the search performance of the neighborhood structure, the study identifies and analyzes optimization strategies for batch partitioning and sequencing, and incorporates an adaptive neighborhood structure adjustment strategy. A competition mechanism is also designed to enhance the algorithm’s optimization efficiency. Simulation experiments of varying scales demonstrate the effectiveness of the variable batch partitioning strategy, showing a 5–6% improvement over equal batch strategies. Results across different scales and parameters confirm the robustness of the algorithm.

## 1. Introduction

With evolving market demands and increasing competition, industries face growing pressure for shorter product delivery times and higher customization requirements. The manufacturing sector, particularly in processing and assembly-oriented enterprises, increasingly emphasizes producing multiple varieties in small batches, often facilitated by batch flow technology [1].

As the diversity and complexity of product types escalate, the need for the coordinated production of parts, components, and products becomes more critical. This complexity often results in extensive buffer areas within workshops for parts turnover and storage, substantially lengthening waiting times throughout the production cycle. Addressing these inefficiencies is vital for boosting productivity per unit area and addressing land scarcity issues in manufacturing settings.

Products such as vacuums, air conditioners, and refrigerators, which exhibit a wide variety in type, generally undergo a three-stage processing and assembly sequence: starting with plastic injection molding, followed by component assembly, and concluding with final product assembly once all parts are ready. These stages are subject to interdependent assembly requirements [2], which can cause delays between stages and lead to the accumulation of work-in-progress in buffer zones, thereby extending production cycles. Therefore, developing efficient scheduling methods is essential for optimizing the production flow of parts and products.

The variability in processing times across different operations and the involvement of multiple machines per process necessitate effective batch division and the strategic allocation of processing equipment to each sub-batch [3]. This study originates from the production processes of multi-variety, small-batch household appliances such as vacuums and aims to explore the three-stage batch flow mixed assembly flow shop scheduling problem, considering non-sequential setup times and multiple tooling constraints, with the goal of minimizing the maximum completion time. Setup times for switching between injection molding molds and assembly jigs primarily occur during setup and debugging phases, independent of production sequence, thus diminishing the sequence’s impact on setup times. Batch flow scheduling [4] is advantageous, as it reduces waiting times, minimizes buffer area sizes, boosts production efficiency, shortens delivery times, and increases output per unit area, rendering it highly relevant and beneficial in practical settings. Existing research on batch flow scheduling often neglects the consideration of production resources beyond equipment, such as raw materials, manpower, and tooling [5]. Different parts frequently require varied molds, and distinct products need specific assembly jigs. The widespread use of molds across diverse industries, such as machinery and electronics, under the multivariety, small-batch production mode, underscores the practical significance of scheduling mixed batches under multiple tooling constraints. In terms of workshop scheduling, the maximum completion time metric effectively captures other conventional scheduling metrics [6], such as waiting times for parts processing, making it the focal optimization objective.

Past research has tackled batch flow mixed assembly flow shop scheduling problems [7,8,9,10,11,12], yet most studies have presumed that sub-batches are either unmixable or of equal size, which does not reflect the complexities of actual production settings. This study addresses more intricate scenarios involving varying sub-batch sizes and multi-stage batch flow systems, aiming to minimize the maximum completion time through an effective migrating birds optimization (EMBO) algorithm.

Moreover, virtual simulation offers substantial potential for production control by facilitating the precise management of complex mixed flows via real-time data interaction. This technological framework ensures the seamless integration of the algorithmic solutions proposed in this study with real-world applications [13,14,15].

The encoding mechanism is structured in two parts to achieve this goal. Its features are utilized to design multiple neighborhood structures incorporating an adaptive adjustment strategy. This strategy improves the search efficiency of the neighborhood structures. Furthermore, to facilitate the verification of the algorithm’s feasibility and to make its application more visual, the algorithm is embedded into a simulation system for implementation. And a data interaction method and the relevant interfaces are designed.

The paper is structured as follows. Section 2 introduces the research status in four related directions, with each sub-section focusing on a specific aspect. Section 3 discusses the system development process, with Section 3.1 describing the overall system design. Section 3.2 elaborates on the problem and its mathematical modeling. Section 4 covers the algorithm design, building on previous content. Section 4.1 discusses the batch strategy, Section 4.2 outlines the encoding and decoding methods, and Section 4.3 explains the improvement strategies and overall algorithm flow. Section 5 addresses the encapsulation of the algorithm and problem, detailing the design process of the visual simulation experiment. Each sub-section includes real-world case studies, experimental parameter settings, algorithm testing, and model comparisons. Finally, Section 6 offers the conclusion.

## 2. Research Background

### 2.1. Virtual Simulation

As orders become increasingly diversified and complex, the demand for refined control within factories has become more apparent. Virtual simulation technology has emerged as a critical solution to meet this demand. Technologies such as digital twins and Cyber–Physical Systems (CPSs) are integral components of this approach [16].

Virtual simulation, as a type of virtual technology, fundamentally represents the actual operation of factories through digital means. The demand for digital twins in industrial production is escalating, driven by their ability to enhance data analysis, regression, and prediction techniques, as well as control processing equipment via information feedback [17]. With advancements in protocols and standards such as OPC UA (Open Platform Communications Unified Architecture), coupled with a growing range of communication technologies, factory digitalization is becoming increasingly achievable [18,19]. Since being introduced in 2012, digital twins have captured significant academic and industrial interest [14]. A robust body of literature has evolved, focusing on developing more reliable and secure digital twin systems and presenting a variety of mature methodologies [17,20,21]. Furthermore, the application of sophisticated algorithms within digital twins and the Industrial Internet of Things (IIoT) has shown considerable promise. Notably, an innovative alternating optimization algorithm has been designed to address mixed-integer non-convex optimization problems in digital twins, aiming to minimize the total task completion delay for all IIoT devices [13]. The adoption of advanced computational techniques such as deep learning and federated optimization learning, which facilitate rapid problem-solving and decision-making, is now extending into areas like energy optimization [14,15].

Additionally, virtual simulation is frequently employed in training programs across various industries, offering a more cost-effective approach. This includes applications in the precast concrete industry [22], training for medical and nursing students [16,23], and psychological education systems [24], among others. Its wide-ranging utility spans across multiple sectors.

### 2.2. Batch Flow Mixed Flow Workshop Scheduling Problem

The batch flow mixed assembly flow shop scheduling problem has been extensively studied. Zhang et al. [7] analyzed a two-stage mixed assembly flow shop with non-interchangeable sub-batches, proposing heuristic algorithms for scheduling sequences and sub-batch divisions with a single device in the second stage. Defersha et al. [8] employed a parallel genetic algorithm for multi-stage mixed assembly flow shop scheduling with interchangeable sub-batches, maintaining uniformity across all processing batches. Zhang et al. [9] enhanced the migrating birds optimization algorithm to tackle equal-sized sub-batch scheduling issues. Qin et al. [10] introduced a two-stage ant colony algorithm focused on multiple constraints for equal-sized sub-batches, while Zhang Biao [11] developed a multi-objective optimization model for multi-variety, small-batch production in both static and dynamic environments. Aqil et al. [12] applied a discrete wavelet optimization algorithm to address the complexities of non-interchangeable sub-batch scheduling. Despite the extensive research, most studies have been predicated on assumptions of non-interchangeable or uniformly sized sub-batches. However, real-world production environments often feature scenarios such as multiple devices processing identical workpieces, diverse batch sizes, and varied batching strategies across different stages. These factors introduce greater complexity and uncertainty into batch division, necessitating more sophisticated scheduling solutions. Although exact algorithms can theoretically provide optimal solutions, their practical application is limited by escalating computational demands as the problem scale increases. In response to these challenges, this study tackles a three-stage batch flow mixed assembly flow shop scheduling problem, considering non-sequential setup times and multiple tooling constraints. It aims to model the problem with the goal of minimizing the maximum completion time, introducing an effective migrating birds optimization algorithm (EMBO) specifically adapted to these complex requirements.

### 2.3. Bionic Optimization Algorithm

The application of bionic optimization algorithms in industrial production has reached a high level of maturity. However, it is crucial to select the appropriate framework for different problems to achieve faster and more effective solutions. Numerous algorithmic frameworks exist in this field, which can be utilized to address various industrial production challenges.

For instance, in their study, M. Rojas-Santiago et al. employed the Ant Colony Optimization (ACO) algorithm to find initial solutions, which were then optimized using other local heuristic algorithms [25]. G. Rivera et al. used the particle swarm optimization (PSO) algorithm to solve scheduling problems involving parallel machines [26]. In the research conducted by O. Abdolazimi et al., an improved Artificial Bee Colony (ABC) algorithm was applied to tackle a newly proposed problem, solving a mathematical model related to Benders decomposition and Lagrangian relaxation algorithms [27]. G. Deng et al. optimized factory production lines using the migrating birds optimization (MBO) algorithm, which incorporates a diversification mechanism, demonstrating a significant advantage over other heuristic algorithms in addressing this issue [28]. Additionally, C. Liu et al. introduced a hybrid algorithm that combines dynamic principal component analysis with a genetic algorithm (GA) for fault diagnosis in industrial processes [29].

As a result, a wide array of bionic optimization algorithms has been applied in the industrial domain, with most achieving commendable success.

Besides biomimetic optimization algorithms, other computational frameworks have proven to offer distinct advantages. Specifically, deep learning and reinforcement learning are emerging as key approaches in this domain.

Shu Luo et al. proposed a multi-layer Deep Q-Network (DQN) agent, which implies that the model can adapt to more complex and urgent rules. This includes a set of six requirements that conform to the rules [30]. In deep reinforcement learning, Deep Q-Networks (DQN) are widely used. Similar studies include Hua Gong’s research on the Flexible Flow Shop Production Scheduling Problem [31] and Yuandou Wang’s work addressing multi-workflow completion time and user costs [32]. In addition to the classic and universal DQN, Gelegen Che and colleagues proposed the use of deep reinforcement learning to address multi-objective optimization problems encountered in production [33]. This approach also facilitates real-time analysis and flexible balancing between cost and performance.

Though reinforcement learning and deep learning deliver strong performance, they typically demand extensive data and computational resources. This makes them less suited for the present study, as gathering such datasets would involve considerable extra costs. Biomimetic optimization algorithms, in contrast to non-biomimetic ones, can yield satisfactory results with fewer resources, making them advantageous for enterprises.

However, biomimetic optimization algorithms still come with high computational costs. Moreover, the solutions from these heuristic algorithms can be unstable, as they mimic biological behaviors, which may not suit all problems. These methods also often demand significant parameter tuning. Despite these challenges, given the need for high performance in multi-modal, non-linear, and complex spaces, these algorithms are better suited for this study. To overcome these limitations, this study introduces several enhancements, essential for ensuring stable and high-quality outcomes.

### 2.4. Modeling and Simulation

Utilizing a physical simulation model is essential for verifying the outcomes of algorithms, ensuring their feasibility by considering a broad spectrum of practical factors. The frameworks and systems dedicated to simulation and modeling are robust, incorporating a variety of emerging simulation technologies [34] and adhering to established simulation standards [35,36].

In the industrial sector, these applications are prevalent. With advancements in real-time communication, simulations have become more aligned with the progress of digital twins, where the interoperability of standards plays a critical role [37].

### 2.5. Research Content

This study focuses on addressing the scheduling challenges in mixed batch flow assembly shops. To tackle this, we first introduce a visual simulation framework for the assembly shop in Section 3, where the problem is modeled and mathematically defined. In Section 4, building on the previous chapter’s mathematical model, we design an MBO algorithm. The algorithm specifically tackles the batch division issue, with tailored strategies designed to address this challenge. The algorithm is then detailed, including its encoding, decoding, evolutionary strategies, and operational processes.

This algorithm is integrated into the visual simulation, with experimental parameters defined accordingly. Comparative simulation experiments with different parameter settings demonstrate the algorithm’s effectiveness in solving the problem, along with the robustness of the simulation framework.

## 3. Virtual Simulation for Assembly Workshop

### 3.1. Design of Virtual Simulation Optimization Systems

**Figure 1:**
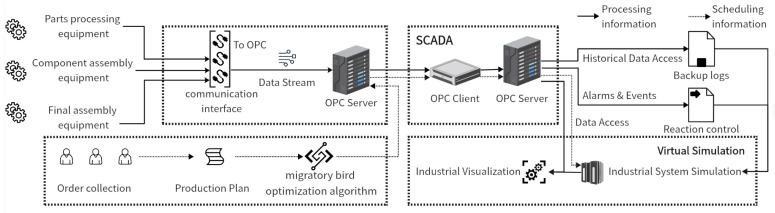
Construction of virtual simulation framework.

This study introduces a virtual simulation framework that leverages a migratory bird optimization algorithm for production scheduling (Figure 1). The production process is monitored using OPC, which facilitates the creation of a comprehensive virtual simulation model. As illustrated in the diagram, various processing and assembly equipment are connected through their respective interfaces to support OPC, enabling effective data transmission. The data from the OPC server are standardized and streamed to the SCADA system’s client. Additionally, order information, collected manually, is utilized to generate a production plan that is subsequently optimized using the migratory bird algorithm. The optimization results are then transmitted to the SCADA system.

Within the SCADA framework, the OPC server administers Historical Data Access, Alarms and Events, and Data Access under three different standards. Data Access is particularly used for making adjustments in the simulation model. The finalized and accurate simulation is then presented on the Industrial Visualization platform.

### 3.2. Problem Description and Modeling

#### 3.2.1. Problem Description

**Figure 2:**
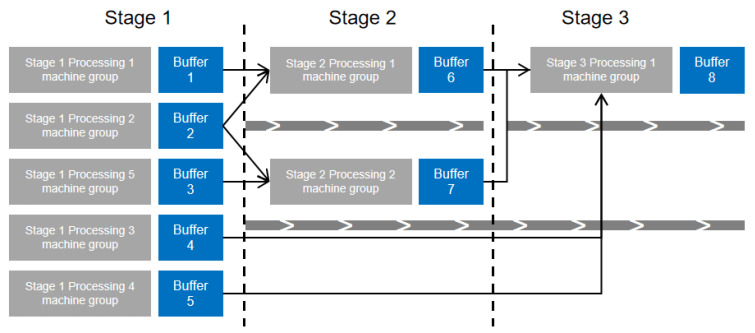
Simplified product process diagram of household appliances.

**Figure 3:**
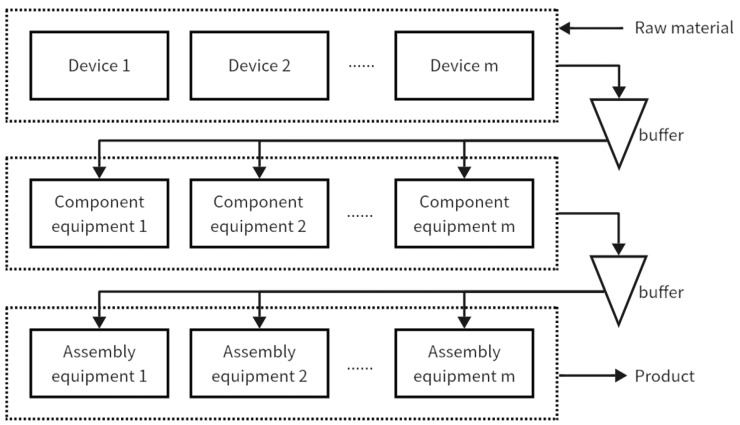
Simplified model diagram of assembly workshop.

This section addresses the challenges associated with multi-variety, small-batch production in product manufacturing. Using principles of product modularization, multiple processing steps for workpieces are streamlined into assembly line configurations. This methodology breaks down the assembly of complex products into distinct, manageable stages. Each stage of a complex product’s assembly, modularized as a single process, is illustrated in the simplified process diagram shown in Figure 2.The focus of this study is a three-stage mixed assembly flow shop, outlined in Figure 3, which includes four primary components: Part 1 involves part machining, where various parts are processed in batches, with different sub-batches handled on multiple unrelated parallel machines. The arrows in the figure indicate the direction of flow of the processed product. The machining time for each part depends on the equipment selection, influenced by setup times and the number of molds. In this study, “mold” refers to injection mold equipment, which serves as a resource occupied during processing. Henceforth, it will be referred to as “mold”. Part 2 encompasses component machining, where assembled parts proceed in batches, unaffected by setup times or mold constraints once the related parts are assembled. Part 3 involves final product assembly, where all components are assembled in batches once they are fully matched. Sub-batches of different products are processed on multiple unrelated parallel machines, subject to setup times and mold quantities. Part 4 serves as a buffer zone, storing incomplete components until parts and products are fully matched for processing and assembly. The workshop manages P types of products, and each stage encompasses Sg processes, with demand quantities Qc per product. Each product’s processing stages correspond to specific mold quantities Ogi across M machines. The primary optimization challenge is to process each stage of all products efficiently while adhering to the mold quantity constraints, and to determine the optimal processing equipment and sequence for each stage’s sub-batches to minimize the maximum completion time.

#### 3.2.2. Problem Modeling

To effectively analyze the problem, the study is based on the following assumptions:

- Setup times are unrelated across different process sequences.
- The buffer zone capacity is considered unlimited.
- The production environment is assumed to be stable, with no consideration for equipment failures or nonconforming production.
- Each sub-batch must be processed continuously on a designated machine without interruption.

To enhance clarity, specific symbols are defined to represent the components of the problem as outlined in Table 1, Table 2 and Table 3.

The objective function aims to minimize the maximum product completion time. This is established through the following conditions:

(1) $$Z=\min\left\{\max_{g=3}\left\{t_{\mathrm{end}}\left(m,K_{k}\left(g,i\right)\right)\right\}\right\}$$

When each sub-batch of a process is less than or equal to the total of corresponding mold and equipment numbers, the expression is formulated as

(2) $$K\left(g,i\right)=\min\left\{O_{S_{g,i}},M_{S_{g,i}}\right\}$$

The total available components for a process equal the remaining components after applying the production and assembly constraints from two-stage sequences:

(3) $$N\left(i,u,v\right)=\sum_{u=1}^{|\xi_{g}|}\sum_{k=1}^{|K_{i}|}\lambda_{k}\times \alpha \left(k,u\right)-\sum_{v=1}^{|\xi_{g}|}\sum_{l=1}^{|K_{i}|}\lambda_{j,l}\times \alpha \left(j,v\right)\times \epsilon \left(i,j\right)$$

The sum of sub-batches for any process equals the total demand across all products corresponding to that process:

(4) $$\sum_{l=1}^{|K_{i}|}\lambda_{k}=\sum_{c=1}^{\left|P\right|}Q_{c}\times W\left(P_{c},S_{g,i}\right)$$

Each sub-batch of any process can only be processed on one machine:

(5) $$\sum_{m=1}^{\left|M\right|}\gamma \left(m,k\right)=1$$

The machine assigned to process each sub-batch of any process belongs to the set of available machines for that process:

(6) $$m_{k}\in M_{i}$$

The relationship between the completion time and processing time of each sub-batch of any process is defined:

(7) $$\begin{array}{cc} t_{end} & (m,K_{k}\left(g,i\right))=\\ \; & t_{start}\left(m,K_{k}\left(g,i\right)\right)+\sum_{m=1}^{M}\gamma \left(m,k\right)\times \lambda_{k}\times t\left(m,S_{g,i}\right)) \end{array}$$

The waiting time constraint between two sub-batches processed consecutively on the same equipment is considered:

(8) $$\begin{array}{cc} t_{start} & (m,K_{k}\left(g,j\right))\geq \\ \; & t_{end}\left(m,K_{k}\left(g,j\right)\right)+t^{\prime }\left(m,S_{g,j}\right))-\left(1-\zeta \left(m,k,l\right)\right)U \end{array}$$

A sub-batch of the product can only start processing if the available component quantity at the current stage is zero or more. The model’s validity and effectiveness have been confirmed through comparison with the CPLEX results:

(9) $$\begin{array}{cc} t_{start}\left(m,K_{k}\left(g,j\right)\right) & +(2-\beta (i,u,v)-\epsilon (i,j))U\\ \; & \geq \max_{u^{\prime }\in \left[1,u+1\right]}\;t_{end}\left(m,K_{k}\left(g,i\right)\right)\times \alpha \left(k,u^{\prime }\right) \end{array}$$

Based on the above summary, the following form of a constraint programming model can be presented:

$$\begin{array}{cc} & \mathrm{Objective}\;\mathrm{function}:\;Z=\min\left\{\max_{g=3}\left\{t_{\mathrm{end}}\left(m,K_{k}\left(g,i\right)\right)\right\}\right\}\\ & \mathrm{Subject}\;\mathrm{to}:\;\mathrm{Equations}\;(2)–(9) \end{array}$$

## 4. Algorithm Design

Given the strong NP-hard characteristics of the batch flow mixed assembly line scheduling problems, precise algorithms often struggle to solve them efficiently within a reasonable timeframe. Intelligent optimization algorithms, tailored to the specific characteristics of these problems, offer a practical approach for rapid solution generation. The migrating birds optimization (MBO) algorithm, a novel metaheuristic with unique sharing and benefiting mechanisms [38], facilitates detailed neighborhood searches and promotes rapid evolution toward optimal solutions. It is renowned for its efficient local search capabilities and excellent convergence performance.

The MBO algorithm has demonstrated superior solution quality in bipartite matching problems [39] and has achieved notable success in addressing scheduling optimization challenges [38,40,41]. In the context of batch scheduling, which requires the careful consideration of batch partitioning and sequencing of sub-batches, the MBO algorithm employs mutation-based neighborhood searches [41]. Its effective exploration of neighborhoods ensures the optimization of batch vectors without violating constraints, rendering it particularly suitable for addressing these complex scheduling issues.

Building on this foundation, we propose an enhanced migrating birds optimization (EMBO) algorithm to tackle the three-stage batch flow mixed assembly line scheduling problem. First, the approach begins with the determination of batch partitioning strategies, which are influenced by mold quantities and collaborative production across the three stages. Second, coding and decoding mechanisms are developed, reflecting the specific characteristics of the problem and the initial partitioning strategies. Next, neighborhood structures specifically tailored for the dual challenges of batch partitioning and sub-batch sequencing are crafted. Finally, strategies for the adaptive adjustment of neighborhood structures and enhancements through competitive mechanisms are introduced to boost the algorithm’s performance. The detailed workflow of the EMBO algorithm is outlined, providing a comprehensive overview of its implementation.

### 4.1. Batch Partitioning Strategy

Common batch partitioning strategies [42] include equal-sized batches, where tasks are divided into equally sized production sub-batches, and unequal-sized batches, where sub-batches may vary in size. These strategies can be further categorized into uniform sub-batches, where the batch partitioning scheme remains consistent across all operations of a task, and variable sub-batches, where the partitioning scheme may differ across different operations of a task. Given the collaborative production across three stages, each with varying processing capacities, adopting different batch partitioning strategies for each stage can effectively minimize the maximum completion time. Consequently, an unequal and variable strategy is adopted: the maximum batch size for each stage operation is determined as $\min\{K\left(g,i\right),M_{S_{g,i}}\}$.

### 4.2. Encoding and Decoding Mechanism

**Figure 4:**
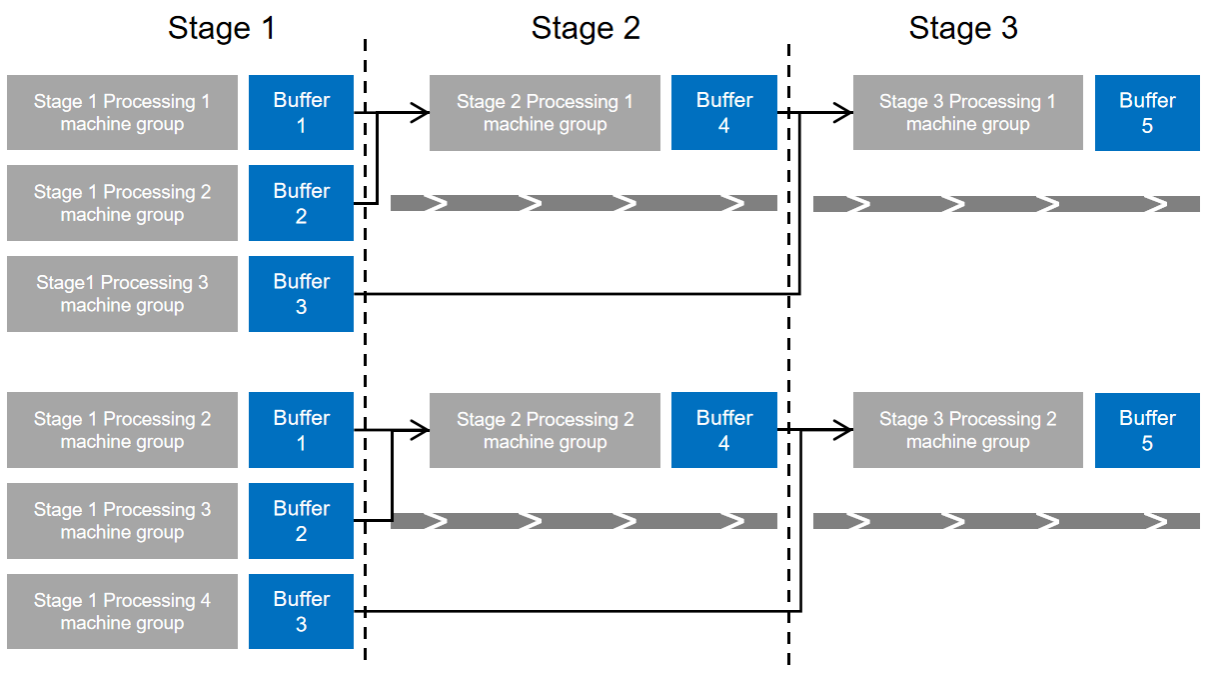
Product three-stage process diagram.

A complete batch flow scheme encompasses the number of batches for each sub-batch, the quantity of each sub-batch, the processing sequence of sub-batches, and the processing equipment for each sub-batch. Addressing the dual sub-problems of batch partitioning and sub-batch scheduling, a two-stage encoding mechanism is devised: batch partitioning encoding and arrangement encoding [43]. To illustrate the encoding mechanism, we use simple Products 1 and 2 as examples. Different operations within the same stage are represented numerically, with specific operations depicted in Figure 4. The maximum number of batches for each stage operation is set at two.

#### 4.2.1. Algorithm Encoding

**Figure 5:**
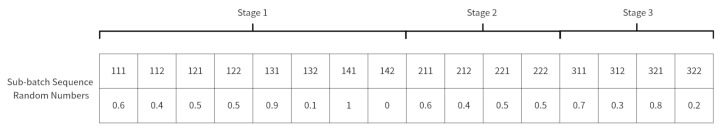
Batch division code generation.

Batch partitioning encoding tackles the number of batches per sub-batch and the quantity of each sub-batch. Utilizing an unequal and variable strategy for batch partitioning necessitates defining the workpiece partitioning schemes for all products across various stages and operations, with varying quantities of workpiece sub-batches, thus escalating the problem’s complexity. To streamline this, identical processing operations for different products are segmented into workpiece sub-batches, facilitating batch partitioning and reducing the computational overhead. For this purpose, we utilize K(g,i) random numbers $X_{i,k}$ with a precision of 0.1 within the range [0, 1.0] to determine the number of batches and the quantity of each sub-batch for operations in all three stages as illustrated in Figure 5.

**Figure 6:**
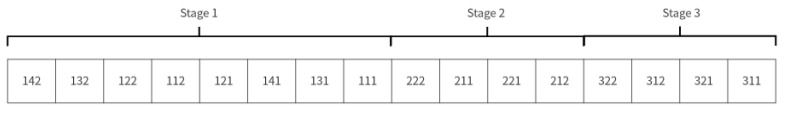
Initial arrangement code generation.

Arrangement encoding focuses on the sub-batch processing sequence. In batch partitioning encoding (Figure 5), sub-batch sequences across different stages are randomly arranged to create the arrangement encoding, addressing the sub-problem of sub-batch scheduling as depicted in Figure 6.

A minimum limit constraint is imposed on sub-batches, requiring that the quantity of each sub-batch either exceeds the minimum production batch size for the corresponding operation or is zero. Equations (10) and (11) are utilized to ascertain the quantity of each sub-batch while adhering to the aforementioned constraint:

(10) $$\lambda_{k}=\left\lfloor \frac{X_{i,k}\times \sum_{c=1}^{\left|P\right|}D_{c}\times W\left(P_{c},S_{g,i}\right)}{\phi_{S_{g,i}}}\right\rfloor \times \phi_{S_{g,i}};\;k<K^{\prime }$$

Here, $K^{\prime }$ represents the actual total number of batches for stage *g*, operation *i*, which is derived from the count of non-zero random numbers $X_{i,k}$. $X_{i,k}$ is a random number between [0, 1.0] for the *k*-th sub-batch of stage *g*, operation *i*:

(11) $$\lambda_{K^{\prime }}=\sum_{c=1}^{\left|P\right|}Q_{c}\times W\left(P_{c},S_{g,i}\right)-\sum_{k^{\prime }=1}^{K^{\prime }-1}\lambda_{k^{\prime }}$$

Here, $\lambda_{K^{\prime }}$ denotes the quantity of the $K^{\prime }$-th sub-batch for stage *g*, operation *i*.

#### 4.2.2. Algorithm Decoding

The decoding mechanism is designed to address the assignment of processing equipment to sub-batches, taking into account the differing capabilities of each piece of equipment. Sub-batches are scheduled based on a “first come, first served” principle. Initially, the “earliest available” rule is applied to determine the most immediate availability of equipment. This is followed by the “capability priority” rule, which prioritizes equipment based on shorter processing times, thereby selecting the most suitable processing equipment from the available set.

By adhering to these rules, alongside considering assembly completion constraints, the start and end times for processing each stage’s sub-batches are established. The ultimate goal of this process is to minimize the maximum completion time, ensuring the efficient and effective use of resources.

### 4.3. Improvement Strategy

#### 4.3.1. Neighborhood Structure

The MBO algorithm evolves by iterating over a neighborhood solution set, where the design of the neighborhood structure significantly impacts the solution quality and convergence speed. Therefore, developing efficient neighborhood structures is essential. The two-stage encoding addresses different sub-problems, each requiring a tailored encoding design for specific neighborhood structures, and introduces a structure that concurrently addresses both sub-problems.

**Figure 7:**
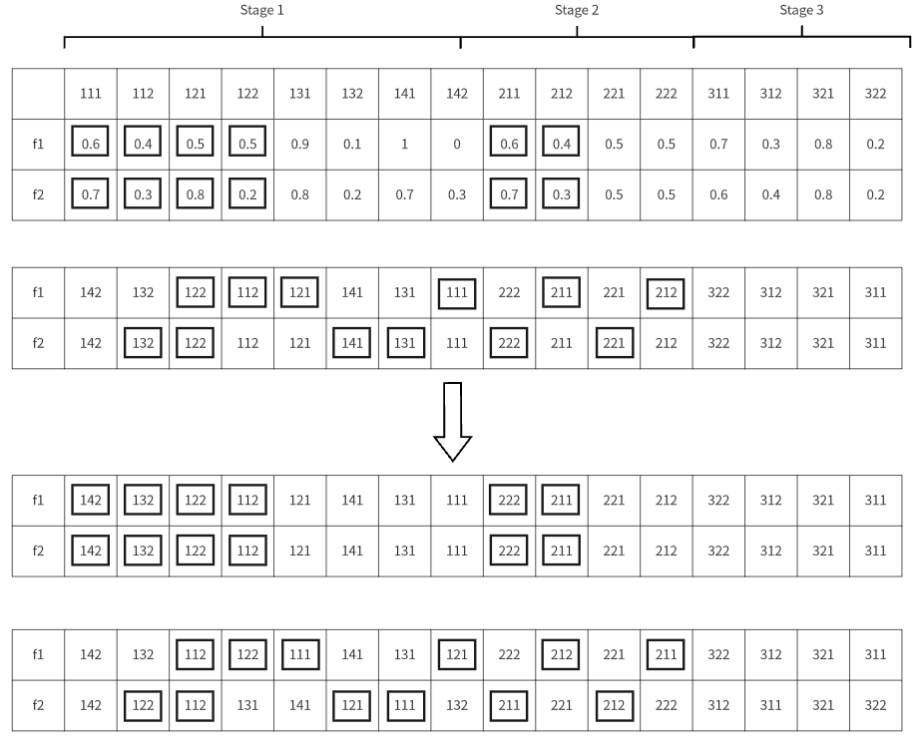
Domain structure transformation of two-segment coding.

(1) Neighborhood Structure for Batch Division Encoding: This involves the mutation of process batch blocks by randomly selecting a sub-batch of a process type at any stage within the constraint range and varying it randomly, which impacts other sub-batches. The number of sub-batches can mutate to zero, effectively reducing the batch count. The exchange of process batch blocks entails randomly selecting two process batch numbers with maximum batch counts for exchange.
(2) Neighborhood Structure for Permutation Encoding: In algorithms for scheduling processing sequences, operations commonly used to construct the neighborhood structure include random exchange, forward insertion, backward insertion, pair exchange, optimal insertion, and optimal exchange.
(3) Neighborhood Structure for Two-Stage Encoding: In the MBO algorithm, early evolution stages with flocking birds are prone to replacement by shared domain solutions, leading to the potential discarding of superior encoding segments. To address this, new neighborhood solutions are proposed by crossing current flocking birds with domain solutions. Considering assembly constraints, a neighborhood structure based on the uniform crossing of subordinate process nodes is proposed. This design involves randomly selecting a node process, forming a set with its subordinate processes, and exchanging random numbers of all process sub-batches in this set. Subsequently, the permutation encoding of all process sub-batches in different stages is exchanged in sequence. For instance, taking Stage 2, Process 1 as an example, new encodings z1 and z2 are generated from the f1 and f2 encodings as detailed in Figure 7.

#### 4.3.2. Adaptive Adjustment of Domain Structure

The algorithm incorporates a total of nine neighborhood structures, each exhibiting varying levels of search effectiveness at different stages of the optimization process. For example, in the later stages, the optimal insertion and optimal exchange structures in the permutation encoding tend to outperform the other four structures, indicating a need to increase their application frequency. To address this, an adaptive adjustment strategy is introduced [44], which updates the corresponding weights based on the performance of neighborhood structures in previous iterations. This adjustment controls the usage probability of each structure through a roulette wheel selection method, optimizing the efficiency of the algorithm across different iterations.

Given the unequal number of neighborhood structures corresponding to the two-stage encoding, a weight of one is assigned to each structure in permutation encoding, and a weight of two to the other structures. The roulette wheel method is used to randomly select a neighborhood structure based on weight $w_{i}$ to generate neighborhood solutions, updating weights after each iteration. The neighborhood structure weights are adjusted using the following formula:

(12) $$w_{i,seg+1}=\left(1-\eta_{i}\right)w_{i,seg}+\eta \frac{X_{i,seg}}{\delta_{i,seg}}$$

where seg denotes the iteration count of the algorithm; $\eta_{i}$ represents the usage frequency of structure *i*; $X_{i}$ accumulates scores for structure *i*, incremented by 1 if structure *i* produces a solution superior to the original; and $\eta_{i}\in \left[0,1.0\right]$ controls how quickly the weight $w_{i}$ responds to the effectiveness of structure *i*.

#### 4.3.3. Competition Mechanism

In the MBO algorithm, after a predetermined number of rounds, the following birds replace the leading birds. If superior solutions are positioned further back in the queue, they may take longer to exert their influence, potentially impeding the optimization efficiency. Furthermore, a lack of interaction between the adjacent queues can decrease the population diversity. To mitigate these issues, intra-population competition is introduced [41] after bird flocking concludes. This strategy helps maintain population diversity and enhances the algorithm optimization efficiency. The specific steps are as follows:

(1) Randomly select a pair of individuals from the following birds and compare their fitness.
(2) If the fitness of the leading bird is lower, swap their positions; otherwise, leave them unchanged.

### 4.4. EMBO Process

**Figure 8:**
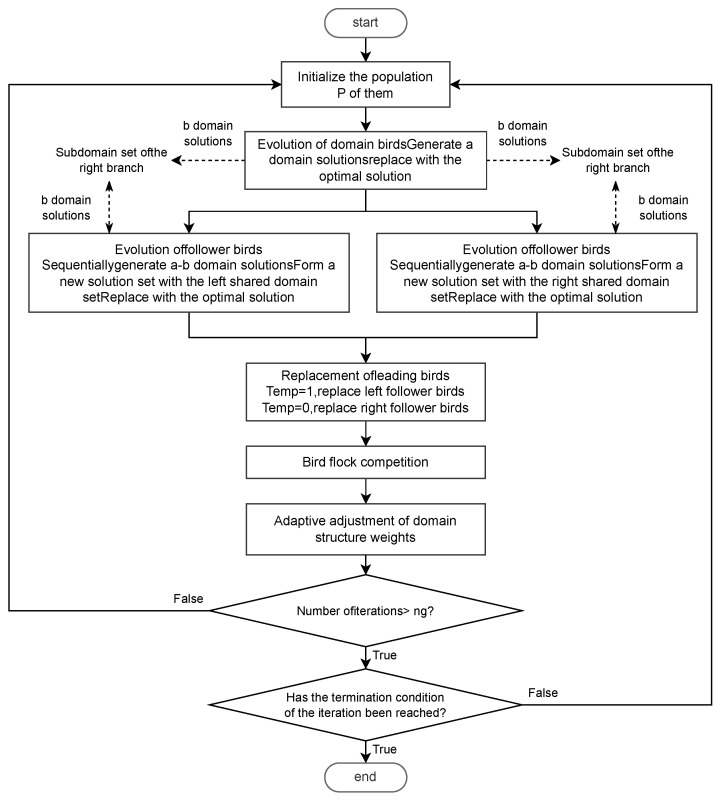
Flow chart of EMBO algorithm.

To facilitate the description of the Enhanced Migrating Birds Optimization (EMBO) algorithm, the following parameters are defined: *N* as the number of individuals in the bird flock, *a* as the number of neighborhood solutions generated by each individual, *b* as the number of neighborhood solutions transferred to the next individual, ng as the number of rounds of migration, Temp as the queue from which the leading birds replace following birds, and $C_{0}$ as the number of competitions. The detailed algorithmic flow of EMBO is illustrated in Figure 8. The diagram consists of two types of lines: dashed and solid. Dashed lines indicate operations on data or solutions, while solid lines illustrate the algorithm’s flow.

## 5. Virtual Simulation Experiment Research

### 5.1. Test Example

**Figure 9:**
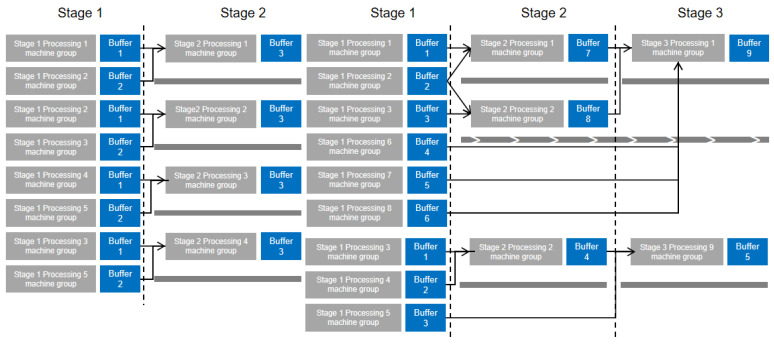
Process drawing of various products.

The testing environment operates on a Windows 10 system equipped with an Intel i7-7700k CPU and 16 GB of RAM, utilizing MATLAB 2016a for programming. As indicated by reference [9], there are no benchmark examples specifically for batch flow shop scheduling. This study utilizes experimental data derived from the production line process of vacuum home appliances at a particular assembly firm, following the methodology outlined by Shao et al. [45]. The experimental parameters are set as follows: the number of product types *P* varies from 3 to 9, with product quantities *W* uniformly distributed between 100 and 400. Stage 2 comprises four types of component operations, while Stage 3 includes four direct part operations. Each product variant involves a varying number of Stage 2 operations *S* (one or two) and Stage 3 operations *S*. The assembly equipment for the final product stage $M_{1}$ ranges from 3 to 7 units, with processing times $t_{1}$ uniformly distributed between 10 and 20 units. Component manufacturing equipment $M_{2}$ includes one to five units, with processing times $t_{2}$ between three and six units. For part manufacturing, the equipment type $M_{3}$ includes one to three units, with processing times $t_{3}$ varying from three to eight units, depending on the equipment type, and some equipment types being unable to process certain parts. Preparation times constrained by the number of molds $t^{\prime }$ are uniformly distributed between 200 and 600 units, with the corresponding number of molds *O* ranging from two to six. The minimum batch size $\phi_{S_{g,i}}$ for each operation stage is uniformly distributed between 20 and 60 units. The notation U[x, y] denotes a discrete uniform distribution between x and y. As shown in Figure 9, different processes can be combined to produce products at different stages.

### 5.2. Parameter Settings

**Figure 10:**
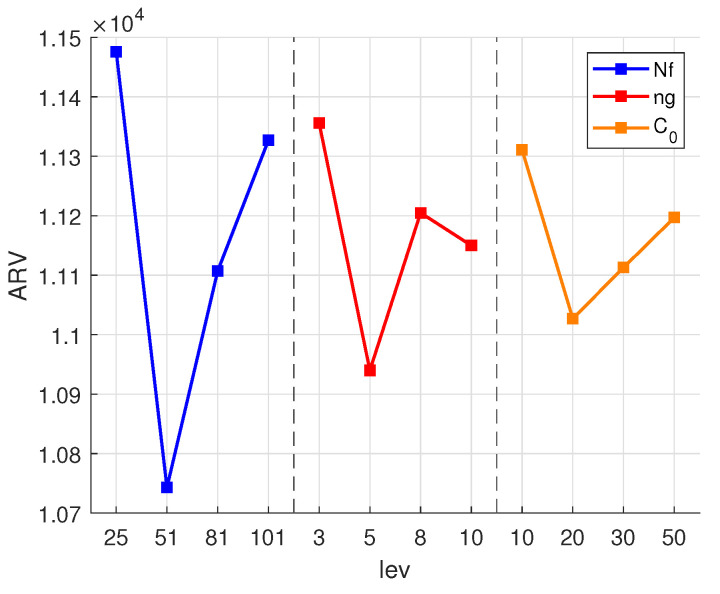
Influence of parameters on performance of EMBO algorithm.

The EMBO algorithm involves parameters *N*, *a*, *b*, ng, and $C_{0}$. Parameters *a* and *b* are mutually constrained and not orthogonal. According to the existing literature and experimental trials, typical settings are a=3 and b=1 owing to their proven effectiveness. Parameters *N*, ng, and $C_{0}$ are determined based on the problem’s scale, utilizing the Taguchi experimental design methods [46]. Factor levels from previous studies, combined with extensive testing, define the parameter settings as shown in Table 4. The algorithm is executed independently 10 times for each parameter combination, with a maximum runtime constraint [11] of $G\times \sum_{g}^{G}\sum_{i}^{S_{g}}K_{i}\times 10S$, where *S* denotes the number of operations in each stage (e.g., $S_{1}$ for Stage 1 operations). Using an example with P=5 and K=67, the average function evaluation *F* serves as the performance metric (ARV). Table 5 presents the results from orthogonal experiments, and Figure 10 depicts trends in the algorithm performance with varying parameters, where “lev” corresponds to levels in Table 4. It is evident that the algorithm performs optimally when N=51, ng=5, and $C_{0}=20$. Subsequent experiments will utilize this parameter combination. Further experiments without an optimal parameter combination yield an ARV of 10,602, demonstrating improved performance compared to previous parameter sets, thereby reaffirming the effectiveness of this parameter configuration. The parameters involved can be represented in Table 6.

### 5.3. Optimality Testing

To validate the model’s accuracy, the EMBO algorithm and CPLEX were independently run 10 times each for solution generation. Given the strong NP-hard nature of the problem, the computational complexity escalates with increasing problem sizes. Due to the lengthy solving times with CPLEX for the aforementioned cases, two scenarios were investigated using EMBO: Product 1, and Products 1 and 2, as shown in Figure 4, with each stage having a maximum batch size of 2. The results were evaluated and compared using the Percent Relative Deviation (PRD) as summarized in Table 7. The data from Table 7 show that in Scenario 1, EMBO produced results identical to those of CPLEX. In Scenario 2, although there was a slight variance in outcomes, the PRD was only 7.3‰, indicating a minimal discrepancy between the two methods and validating the effectiveness of the EMBO algorithm.

### 5.4. Comparison of Batch Modes

**Figure 11:**
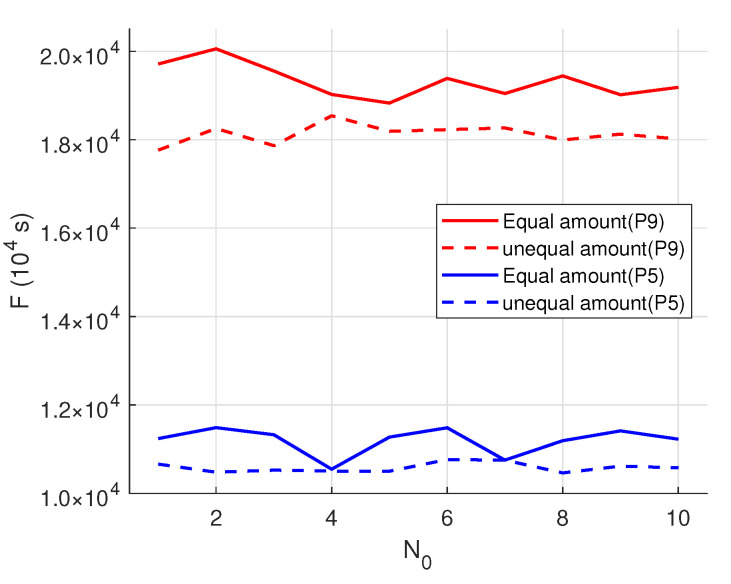
Results of two studies under different batch strategies.

To assess the effectiveness of the variable-sized batch strategy, it was compared against an equal-sized batch strategy across different production scales, product categories, and total maximum batch counts. Two scenarios, P=5, K=67 and P=9, K=78, were selected for analysis. The EMBO algorithm was independently run 10 times for each scenario, and the experimental results are illustrated in Figure 11. In this figure, “No” denotes the experiment number, and “F” represents the minimization of the maximum product completion time. The results clearly demonstrate that the variable-sized batch strategy, by allowing flexible batch division across stages, optimizes the utilization of equipment, thereby reducing equipment idle time and subsequently minimizing the maximum completion time. As the number of products and batches increases, the variable-sized strategy proves to be more effective, outperforming the equal-sized strategy by 5–6% in the test cases.

### 5.5. Algorithm Comparison

To validate the performance of the EMBO algorithm, it was compared against three effective algorithms for solving flexible flow shop scheduling problems: the improved genetic algorithm (IGA) [42], improved particle swarm optimization (IPSO) [47], and improved migrating birds optimization (IMBO) [48]. The original recommended parameter settings from the literature were used for each algorithm. Due to the distinct nature of our research problem compared to those previously studied as detailed in Table 8, the mean optimal solutions of the EMBO and IMBO algorithms significantly outperformed those of the IGA and IPSO algorithms, with EMBO generally providing better mean optimal solutions than IMBO. However, the difference in mean optimal solutions between the two bird-inspired algorithms is small, as is the standard deviation, prompting a *t*-test comparison between the EMBO and IMBO algorithms, presented in Table 9. In most cases, the mean optimal solutions of the EMBO algorithm surpassed those of the IMBO algorithm, highlighting the significant advantage of the EMBO algorithm in the three-stage mixed-model assembly flow shop scheduling problem under the variable-sized batch strategy.

**Figure 12:**
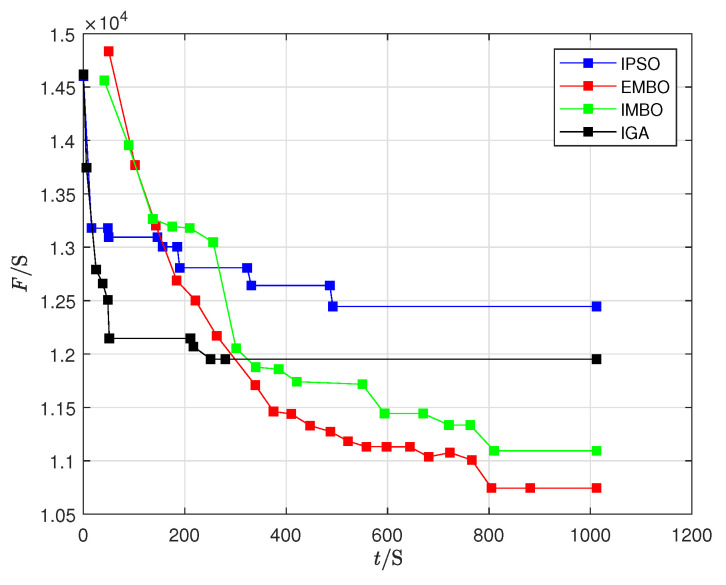
Convergence effect diagram of three algorithms.

Furthermore, the EMBO algorithm typically achieves a smaller Relative Standard Deviation (RSD) compared to the other three algorithms across various total numbers of sub-batches, with the highest RSD being only 1.94%. This demonstrates the stability and robustness of the EMBO algorithm’s optimization results. Using the scenario with P=5, K=67, the convergence and runtime comparisons of the four algorithms are depicted in Figure 12, where *t* represents the CPU runtime and *F* the minimization of the maximum product completion time.

From Figure 12, it is clear that although the IGA and IPSO algorithms converge more quickly within a certain runtime, the EMBO and IMBO algorithms achieve better convergence accuracy. Compared to IMBO, the EMBO algorithm demonstrates faster convergence and is more adept at escaping local optima, further validating its effectiveness.

**Figure 13:**
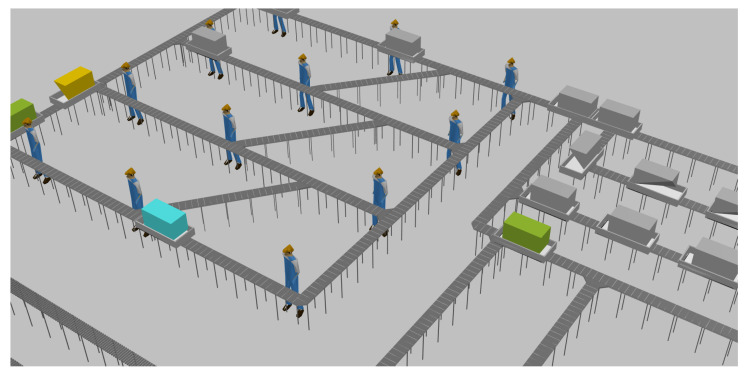
Convergence effect diagram of three algorithms.

Additionally, a physical simulation experiment was conducted using AnyLogic to validate the designed algorithm further. The simulation results confirm that the transportation of most components and finished products is smooth. A part of the factory setup is illustrated in Figure 13.

## 6. Conclusions

For the three-stage hybrid model assembly flow shop scheduling problem, this study, from the perspective of bionic optimization algorithms, simulates the behavior of migratory birds and proposes an EMBO algorithm tailored to address this specific problem efficiently integrated within a virtual simulation framework to enable the real-time monitoring and control of production processes. A comprehensive communication system has been developed based on OPC to support the virtual simulation model. Comparative analyses between non-equal-sized variable batching strategies and equal-sized variable batching strategies demonstrate the superior effectiveness of the former in the context of mixed-model assembly flow shop scheduling. Extensive random repeated experiments on cases of various scales and comparisons with other intelligent algorithms for batch scheduling problems confirm the effectiveness and robustness of the EMBO algorithm in addressing the mixed-model assembly flow shop scheduling problem with non-equal-sized variable batching. The research also involves a detailed investigation into the three-stage mixed-model assembly flow shop scheduling problem, leading to the establishment of a mathematical model that explores batch partitioning strategies within this context. An EMBO algorithm has been designed, featuring a two-phase encoding approach based on batch partitioning and permutation sequence sub-problems. Additionally, a domain structure that simultaneously optimizes these two sub-problems and an adaptive adjustment strategy for multiple domain structures are proposed. The primary optimization objective of this study is to minimize the maximum completion time. Future research will expand the scope to include multiple objectives such as delivery time, production cost, and total flow time to better align with practical production scenarios, thereby further enhancing the investigation into batch scheduling strategies.

This study does not address multi-objective production scheduling, including factors like meeting delivery deadlines, minimizing costs, and reducing total flow time. In practice, these objectives are crucial and warrant further exploration in batch flow scheduling. Future research will focus on addressing these objectives.

## Figures and Tables

**Table 1:** Index and Input Data with Data Types.

Symbol	Type	Parameter	Description
*P*	int	*c*	Vector of product types, indexed by *c*
*D*	int	*c*	Demand quantity for product $P_{c}$
G={G1,G2,G3}	int	*g*	Three production stages
$S_{g}$	int	*i*, *j*	Vector of processes in stage *g*, indexed by *i*, *j*
$W(P_{c},S_{g,i})$	int	*c*, $S_{g,i}$	Quantity of workpieces for product *c* at stage *g*, process *i*
$\phi_{S_{g,i}}$	int	$S_{g,i}$	Minimum production batch for stage *g*, process *i*
$M_{S_{g,i}}$	int	$S_{g,i}$, *m*, *n*	Vector of available equipment for stage *g*, process *i*
$t(m,S_{g,i})$	float	*m*, $S_{g,i}$	Processing time on equipment *m* for stage *g*, process *i*
$t^{\prime }\left(m,S_{g,i}\right)$	float	*m*, $S_{g,i}$	Transition time from current processing to stage *g*, process *i* on equipment *m*
U	long	-	A large positive number
$O_{S_{g,i}}$	int	$S_{g,i}$	Number of molds for stage *g*, process *i*; specifically, $O_{S_{g,i}}=U$ if the current process does not involve molds

**Table 2:** Variables with data types.

Symbol	Type	Parameter	Description
K(g,i)	int	*k*, *i*	Maximum total batches for stage *g*, process *i*
$\xi_{g}$	int	*u*, *v*	Sequence formed by sub-batches of stage *g* processes in a certain processing order; *u* corresponds to *k* and *v* corresponds to *l*, indexed by *u*, *v*
N(i,u,v)	int	*g*, *h*, *u*, *v*, *i*	Total available components for stage *g*, process *i* after processing *u* sequences in stage *g* and *v* sequences in stage *h* (g<h).
$\lambda_{k}$	int	*k*, *g*, *i*	Size of the *k*th sub-batch for stage *g*, process *i*
$m_{k}$	int	*k*, *g*, *i*	Equipment number for the *k*th sub-batch of stage *g*, process *i*
$t_{start}\left(m,K_{k}\left(g,i\right)\right)$	float	*m*, $K_{k}\left(g,i\right)$	Start time of the *k*th sub-batch processing on equipment *m* for stage *g*, process *i*
$t_{end}\left(m,K_{k}\left(g,i\right)\right)$	float	*m*, $K_{k}\left(g,i\right)$	Completion time of the *k*th sub-batch processing on equipment *m* for stage *g*, process *i*

**Table 3:** Boolean variables with data types.

Symbol	Type	True Condition	False Condition
α(k,u)	Boolean	1 if the *k*th sub-batch of stage *g*, process *i* belongs to processing sequence *u*	0 otherwise
β(i,u,v)	Boolean	1 if total available components for stage *g*, process *i* after processing *u* sequences in stage *g* and *v* sequences in stage *h* is less than 0	0 otherwise
γ(m,k)	Boolean	1 if the *k*th sub-batch of stage *g*, process *i* is allocated to equipment $m_{k}$ for processing	0 otherwise
ϵ(i,j)	Boolean	1 if stage *g*, process *i* needs to be processed and assembled before stage *h*, process *j*	0 otherwise
ζ(m,k,l)	Boolean	1 if the *k*th sub-batch of stage *g*, process *i* on equipment *m* needs to be processed before the *l*th sub-batch of stage *h*, process *j*	0 otherwise

**Table 4:** Parameter level table of EMBO algorithm.

Value	Parameter		
	N	ng	$C_{0}$
1	25	3	10
2	51	5	20
3	81	8	30
4	101	10	50

**Table 5:** Results of orthogonal experiments.

Experiment Number	Parameter			ARV
	***Nf***	***ng***	$C_{0}$	
1	25	3	10	11,823
2	25	5	20	11,096
3	25	8	30	11,655
4	25	10	50	11,326
5	51	3	50	10,736
6	51	5	10	10,747
7	51	8	50	10,732
8	51	10	30	10,751
9	81	3	30	11,226
10	81	5	50	11,094
11	81	8	10	11,128
12	81	10	20	10,977
13	101	3	50	11,638
14	101	5	30	10,821
15	101	8	20	11,299
16	101	10	10	11,548
Level 1	11,476	11,356	11,311	—
Level 2	10,743	10,940	11,027	—
Level 3	11,107	11,205	11,113	—
Level 4	11,327	11,150	11,197	—
Delta	734	415	284	—
Sort	1	2	3	—

**Table 6:** Parameter Symbols, Types, and Descriptions.

Symbol	Type	Description
*N*	Integer	Number of iterations in the algorithm
*a*	Integer	Parameter in the EMBO algorithm, typically a=3
*b*	Integer	Parameter in the EMBO algorithm, typically b=1
ng	Integer	Number of generations in the algorithm
$C_{0}$	Integer	Initial constant in the algorithm, typically $C_{0}=20$
*G*	Integer	Scaling factor in runtime constraint
*K*	Integer	Number of operations per stage
*S*	Integer	Number of operations in each stage
*P*	Integer	Problem scale parameter, example value P=5
*F*	Float	Average function evaluation, performance metric

**Table 7:** Comparison between the EMBO algorithm and CPLEX solution %.

	CPLEX	EMBO	PRD
1	6600	6600	0.0
2	8100	8160	7.3

**Table 8:** Statistical results of four algorithms.

Serial Number	Example Calculation		EMBO			IGA			IPSO			IMBO		
	**P**	**K**	**Avg**	**Std**	**RSD**	**Avg**	**Std**	**RSD**	**Avg**	**Std**	**RSD**	**Avg**	**Std**	**RSD**
1	3	55	8272	119	1.44%	9121	256	2.80%	9510	330	3.47%	8675	237	2.73%
2	3	60	8143	125	1.54%	8875	263	2.96%	9283	263	2.83%	8464	240	2.83%
3	3	65	8072	142	1.76%	8787	321	3.66%	9219	229	2.48%	8328	209	2.51%
4	5	67	10,786	135	1.25%	12,018	334	2.78%	12,787	325	2.54%	10,851	234	2.16%
5	5	72	10,637	129	1.22%	11,928	358	3.00%	12,468	255	2.04%	10,753	243	2.26%
6	5	70	10,583	122	1.16%	11,507	367	3.19%	12,403	189	1.52%	10,645	217	2.04%
7	7	73	15,200	122	0.81%	16,374	580	3.54%	18,036	374	2.08%	15,385	140	0.91%
8	7	74	15,046	169	1.12%	16,422	422	2.57%	17,690	196	1.11%	15,200	232	1.52%
9	9	91	14,667	203	1.38%	15,639	442	2.82%	17,534	260	1.48%	14,758	229	1.55%
10	9	78	18,411	159	0.86%	20,303	362	1.78%	21,870	353	1.61%	19,254	144	0.75%
11	9	86	18,220	130	0.72%	19,852	373	1.88%	21,558	374	1.74%	18,835	213	1.13%
12	10	78	18,133	226	1.25%	19,936	366	1.84%	21,545	273	1.27%	18,520	242	1.31%

**Table 9:** Comparison of the optimal solution of EMBO and IMBO algorithms with a confidence level of 0.95.

Example Calculation	EMBO-IMBO	
	**Upper Confidence Limit**	**Lower Confidence Limit**
1	−560	−257
2	−457	−195
3	−392	−130
4	−221	83
5	−247	22
6	−196	77
7	−310	−65
8	−299	−8
9	−214	33
10	−921	−767
11	−766	−470
12	−584	−195

## Data Availability

The original contributions presented in the study are included in the article, further inquiries can be directed to the corresponding author.
